# Recurrence pattern and its predictors for advanced gastric cancer after total gastrectomy

**DOI:** 10.1097/MD.0000000000023795

**Published:** 2020-12-18

**Authors:** Xuguang Jiao, Yu Wang, Feng Wang, Xinbo Wang

**Affiliations:** a Department of Surgical Oncology; b Department of Information Network Management, Weifang People's Hospital; Weifang, Shandong, P.R. China.

**Keywords:** forecasting, gastrectomy, recurrence, stomach neoplasms

## Abstract

This study aimed to investigate the recurrence patterns of advanced gastric cancer (AGC) after curative total gastrectomy and further explore predictors for each pattern of recurrence.

Data of 299 AGC patients between 2010 and 2014 were retrospectively analyzed to investigate the clinicopathologic factors affecting the recurrence pattern of AGC patients underwent curative total gastrectomy.

Sixty-eight (22.7%) AGC patients had recurrence after total gastrectomy. Distant metastasis (DM) was the most prevalent pattern with 29 (42.6%) cases, followed by peritoneal recurrence (PR) with 25 (36.8%) patients, and locoregional recurrence (LR) occurred in 23 (33.8%) patients. The recurrence rates within 2 and 5 years were 77.9% and 97.1%. Extent of lymphadenectomy (*P* < .001, *χ*^2^ = 17.366), depth of tumor invasion (*P* < .001, *χ*^2^ = 21.638), lymph node metastasis (*P* = .046, *χ*^2^ = 9.707), and number of negative lymph nodes (*P* = .017, *χ*^2^ = 2.406) were associated with tumor recurrence by univariate analysis. Multivariate analyses revealed that the extent of lymphadenectomy (*P* = .034, 95% CI: 1.074–6.414) and T4b status (*P* = .015, 95% CI: 0.108–0.785) were independent predictors for LR; histological type (*P* = .041, 95% CI: 0.016–0.920) and T4b status (*P* = .007, 95% CI: 0.102–0.690) for PR; and pN status (*P* = .032) for DM.

In AGC patients following total gastrectomy, recurrent predictors various among locoregional, peritoneal, and distant recurrence. Recurrent predictors of tumor invasion, lymph node metastasis, and histological type could guide follow-up and risk-oriented adjuvant treatment, extended lymphadenectomy was considered to reduce LR of AGC patients after curative total gastrectomy.

## 1 Introduction

Gastric cancer (GC) is the fourth malignant carcinoma with the third highest mortality rate in the world.^[1,2]^ Despite the proportion of early diagnosis and treatment level has improved, the therapeutic effect of GC is still unsatisfactory. Recurrence is the main reason for death of GC patients. The recurrence rate varies considerably between institutions and countries with 21.8% to 50% of patients after curative surgery because most of patients were in an advanced stage at the time of initial visit.^[3,4]^ Furthermore, most of the postoperative recurrence usually occurs within 2 years and is rapidly fatal, thus early recurrence detection and timely treatment intervention are essential.^[5,6]^

The postoperative recurrence patterns of GC are mainly classified as locoregional recurrence (LR), peritoneal recurrence (PR), and distant metastasis (DM). Recurrence pattern varies considerably between tumor locations and stages. Patterns of early disease, advanced disease, proximal gastric cancer, and so on had been reported, while advanced gastric cancer (AGC) after total gastrectomy yet reported.^[7–9]^ Furthermore, depth of tumor invasion, lymph node metastasis, histological type, and some other factors could predict recurrence after surgery.^[10–13]^ Therefore, the current study aimed to reveal the predictors for different recurrence patterns of AGC followed by total gastrectomy.

## 2 Materials and methods

### 2.1 Eligible patients

We reviewed and analyzed 996 patients followed by curative gastrectomy between January 2008 and December 2012 at the Department of Surgical Oncology, Weifang People's Hospital retrospectively.

The eligibility criteria included: pathologically confirmed adenocarcinoma; patients received radical total gastrectomy (R0) with D1 or D2 lymphadenectomy; >15 dissected lymph nodes.

The exclusion criteria included: patients received partial gastrectomy; patients presented with para-aortic lymph node metastasis; patients with distant metastasis or peritoneal dissemination during surgery; patients who were lost to follow-up.

In total, 299 eligible patients were included in the present study.

### 2.2 Clinical and pathological data collection and variable classification

The clinicopathological features studied included sex, age, tumor location, tumor size, histological type, pT stage, pN stage, count of negative lymph nodes, lymphadenectomy, and postoperative chemotherapy.

Recurrences were divided into LR, PR, and DM. LR was defined as the detection of tumors in the gastric bed, within the lymph nodes (including regional, retropaneratic, and para-aortic lymph nodes), or anastomotic sites. PR was defined as any tumor recurrence within the abdominal cavity, such as intraperitoneal distribution. DM included visceral and cutaneous or musculoskeletal involvement of cancer. The independent Ethics Committee of Weifang People's Hospital (Shandong, China) approved this study.

### 2.3 Follow-up

During the first 2 years patients were followed every 3 months. Between 2 and 5 years, follow-up was performed every 6 months. After 5 years patients were followed up once a year until death or January 2017. Laboratory and imaging examinations were performed at every follow-up. Enhancement computed tomography was used to diagnose tumor recurrence when other examination results suggested suspected metastasis. Endoscopy was used for the pathological diagnosis of anastomotic recurrence.

### 2.4 Statistical analysis

Patient characteristics were evaluated using a Student *t* test for continuous data and a chi-squared test for categorical variables in univariate analysis. Multivariate analysis was performed using a binary logistic regression model to identify independent recurrence predictors. *P* < .05 was considered to indicate a statistically significant difference. All statistical analysis was performed using SPSS (version 17.0, Chicago, IL).

## 3 Results

### 3.1 Time and patterns of recurrence for all patients

**Figure 1:**
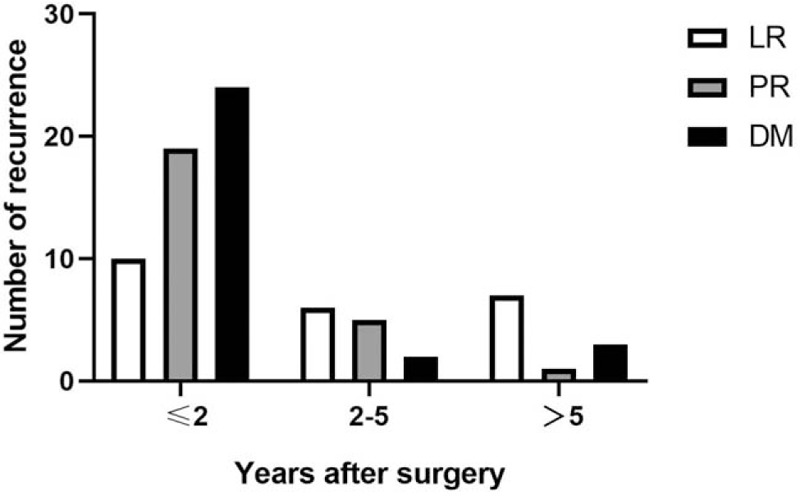
The time of recurrence of AGC patients after total gastrectomy. AGC = advanced gastric cancer; DM = distant metastasis; LR = locoregional recurrence; PR = peritoneal recurrence.

**Figure 2:**
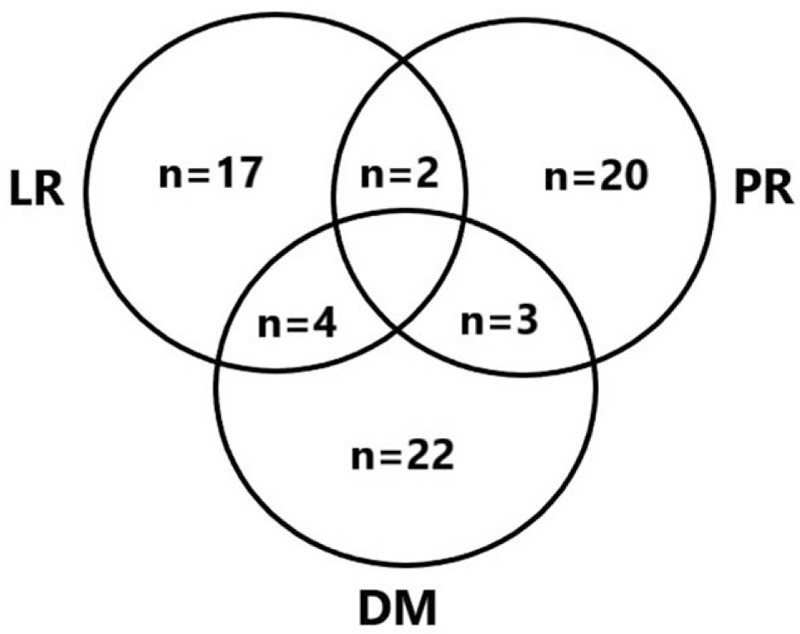
The pattern of recurrence of AGC patients after total gastrectomy. AGC = advanced gastric cancer; DM = distant metastasis; LR = locoregional recurrence; PR = peritoneal recurrence.

Sixty-eight (22.7%) patients presented with recurrence in all 299 patients. The median time to recurrence was 17.7 months (ranged from 3.0 to 74.0 months). The frequency and proportion of recurrent cases, according to the duration after surgery, are shown in Figs. 1 and 2 and Table 1. Fifty-three (77.9%) patients recurred within the first 2 years, 66 (97.1%) patients recurred within 5 years after surgery (Fig. 1). The LR, PR, and DM rate were 43.5%, 76%, and 82.8%, during 2 to 5 years after surgery they were 26.1%, 20%, 6.9%, and after 5 years they were 30.4%, 4%, and 10.3% respectively. Overall 59 patients had a single recurrence pattern, 9 patients had recurrence involving 2 areas, and none had recurrence involving all 3 areas. Twenty-nine (42.6%) patients showed DM, which was the most prevalent, PR occurred in 25 (36.8%) patients, and 23 (33.8%) patients had LR (Fig. 2). Table 1 shows the sites of patients with recurrence, lymph node recurrence (65.3%) was the most frequent component of LR, followed by gastric bed (21.7%) and anastomosis (13.0%) recurrence. Liver (48.3%) was the most common involved organ in DM.

**Table 1:** Sites of 68 AGC patients with recurrence after a curative total gastrectomy.

Site	Cases
Locoregional recurrence	23
Anastomosis	3 (13.0%)
Gastric bed	5 (21.7%)
Lymph nodes	15 (65.3%)
Peritoneal recurrence	25
Distant metastasis	29
Liver	14 (48.3%)
Lung	5 (17.2%)
Ovary	4 (13.8%)
Bone	4 (13.8%)
Brain	2 (6.9%)

### 3.2 Predictors of various recurrence patterns

Table 2 shows the comparison of clinicopathological indexes between recurrent group and non-recurrent group. Among these patients, 68 (22.7%) had tumor recurrence. Extent of lymphadenectomy (*P* < .001), pT stage (*P* < .001), pN stage (*P* = .046), and number of negative lymph nodes (*P* = .017) were found to be the significant risk factors for tumor recurrence in univariate analysis.

**Table 2:** Clinicopathological characteristics of 299 patients with advanced gastric cancer after total gastrectomy in terms of recurrence.

		Recurrence			
Parameter	Total	Yes	No	*χ*^2^	*P*-value
Number	299	68 (22.7)	231 (77.3)		
Gender				0.026	.872
Male	191 (63.9)	44 (64.7)	147 (63.6)		
Female	108 (36.1)	24 (35.3)	84 (36.4)		
Age at surgery, y^∗^	58.1 ± 11.7	56.6 ± 11.9	58.5 ± 11.6	1.183	.238
Tumor size, cm^∗^	6.55 ± 3.01	6.96 ± 3.07	6.42 ± 2.98	1.301	.194
Tumor location				6.295	.098
Upper	80 (26.8)	23 (33.8)	57 (24.7)		
Middle	78 (26.1)	21 (30.9)	57 (24.7)		
Lower	38 (12.7)	4 (5.9)	34 (14.7)		
Diffuse	103 (34.4)	20 (29.4)	83 (35.9)		
Lymphadenectomy				17.366	<**.001**
Limited (D1)	71 (23.7)	29 (42.6)	42 (18.2)		
Extended (D2 or D3)	228 (76.3)	39 (57.4)	189 (81.8)		
Borrmann type				1.074	.353
I+II	82 (27.4)	22 (32.4)	60 (26.0)		
III+IV	217 (72.6)	46 (67.6)	171 (74.0)		
Histological type				1.541	.273
Differentiated	79 (26.4)	14 (20.6)	65 (28.1)		
Undifferentiated	220 (73.6)	54 (79.4)	166 (71.9)		
pT stage				21.638	<**.001**
T2	11 (3.7)	0	11 (4.8)		
T3	28 (9.4)	2 (2.9)	26 (11.3)		
T4a	226 (75.6)	49 (72.1)	177 (76.6)		
T4b	34 (11.4)	17 (25.0)	17 (7.4)		
pN stage				9.707	**.046**
N0	54 (18.1)	6 (8.8)	48 (20.8)		
N1	43 (14.4)	10 (14.7)	33 (14.3)		
N2	73 (24.4)	18 (26.5)	55 (23.8)		
N3a	72 (24.1)	14 (20.6)	58 (25.1)		
N3b	57 (19.1)	20 (29.4)	37 (16.0)		
Number of negative lymph nodes^∗^	17.90 ± 11.61	14.94 ± 11.67	18.77 ± 11.48	2.406	**.017**
Adjuvant chemotherapy				0.550	.481
No	116 (38.8)	29 (42.6)	87 (37.7)		
Yes	183 (61.2)	39 (57.4)	144 (62.3)		

Table 3 shows the relationship between the clinicopathological features and the 3 recurrence patterns by univariate analysis. Type of lymphadenectomy (*P* = .037), pT stage (*P* = .01) were associated with LR. Histological type (*P* = .007) and pT stage (*P* = .002) were 2 risk factors for PR. The pN stage (*P* < .001) and number of negative lymph nodes (*P* = .013) were the predictors for DM.

**Table 3:** Univariate analysis of clinicopathologic factors for different recurrence patterns in patients with AGC after total gastrectomy.

	Locoregional recurrence			Peritoneal recurrence			Distant metastasis		
Parameter	Yes	No	*P*-value	Yes	No	*P*-value	Yes	No	*P*-value
Number	23 (7.7)	276 (92.3)		25 (8.4)	274 (91.6)		29 (9.7)	270 (90.3)	
Gender			.176			.200			.685
Male	18 (78.3)	173 (62.7)		13 (52.0)	178 (65.0)		20 (69.0)	171 (63.3)	
Female	5 (21.7)	103 (37.3)		12 (48.0)	96 (35.0)		9 (31.0)	99 (36.7)	
Age at surgery, y^∗^	59.8 ± 10.9	58.0 ± 11.8	.471	54.8 ± 12.5	58.4 ± 11.6	.142	55.6 ± 12.2	58.4 ± 11.6	.225
Tumor size, cm^∗^	7.37 ± 3.11	6.48 ± 2.99	.172	6.66 ± 3.18	6.54 ± 3.00	.844	7.43 ± 2.90	6.45 ± 3.01	.096
Tumor location			.083			.671			.686
Upper	11 (47.8)	69 (25.0)		5 (20.0)	75 (27.4)		9 (31.0)	71 (26.3)	
Middle	6 (26.1)	72 (26.1)		8 (32.0)	70 (25.5)		9 (31.0)	69 (25.6)	
Lower	1 (4.3)	37 (13.4)		2 (8.0)	36 (13.1)		2 (6.9)	36 (13.3)	
Diffuse	5 (21.7)	98 (35.5)		10 (40.0)	93 (33.9)		9 (31.0)	94 (34.8)	
Lymphadenectomy			**.037**			.139			.065
Limited (D1)	10 (43.5)	61 (22.1)		9 (36.0)	61 (22.3)		11 (37.9)	59 (21.9)	
Extended (D2 or D3)	13 (56.5)	215 (77.9)		16 (64.0)	213 (77.7)		18 (62.1)	211 (78.1)	
Borrmann type			.089			.350			.828
I+II	10 (43.5)	72 (26.1)		9 (36.0)	73 (26.6)		7 (24.1)	75 (27.8)	
III+IV	13 (56.5)	204 (73.9)		16 (64.0)	201 (73.4)		22 (75.9)	195 (72.2)	
Histological type			.806			**.007**			.516
Differentiated	5 (21.7)	74 (26.8)		1 (4.0)	78 (28.5)		9 (31.0)	70 (25.9)	
Undifferentiated	18 (78.3)	202 (73.2)		24 (96.0)	196 (71.5)		20 (69.0)	200 (74.1)	
pT stage			**.010**			**.002**			.666
T2	0	11 (4.0)		0	11 (4.0)		0	11 (4.1)	
T3	0	28 (10.1)		0	28 (10.2)		2 (6.9)	26 (9.6)	
T4a	16 (69.6)	210 (76.1)		17 (68.0)	211 (77.0)		24 (82.8)	204 (75.6)	
T4b	7 (30.4)	27 (9.8)		8 (32.0)	24 (8.8)		3 (10.3)	29 (10.7)	
pN stage			.372			.277			<**.001**
N0	2 (8.7)	52 (18.8)		4 (16.0)	50 (18.2)		0	54 (20.0)	
N1	4 (17.4)	39 (14.1)		5 (20.0)	38 (13.9)		1 (3.4)	42 (15.6)	
N2	5 (21.7)	68 (24.6)		9 (36.0)	64 (23.4)		6 (20.7)	67 (24.8)	
N3a	9 (39.1)	63 (22.8)		2 (8.0)	70 (25.5)		8 (27.6)	64 (23.7)	
N3b	3 (13.0)	54 (19.6)		5 (20.0)	52 (19.0)		14 (48.3)	43 (15.9)	
Number of negative lymph nodes^∗^	13.70 ± 9.97	18.25 ± 11.69	.071	16.96 ± 12.65	17.89 ± 11.62	.703	12.72 ± 11.52	18.36 ± 11.59	**.013**
Adjuvant chemotherapy			.117			.198			.549
No	5 (21.7)	111 (40.2)		13 (52.0)	103 (37.6)		13 (44.8)	103 (38.1)	
Yes	18 (78.3)	165 (59.8)		12 (48.0)	171 (62.4)		16 (55.2)	167 (61.9)	

Multivariate analyses showed that pT stage (T4a/T4b, *P* = .015) and extent of lymphadenectomy (D1/D2, D3, *P* = .034) were independently associated with LR (Table 4). The pT stage (T4a/T4b, *P* = .007) and histological type (Differentiated/Undifferentiated, *P* = .041) were independent predictors for PR (Table 5), and pN stage (N1/ N3b, *P* = .013; N2/ N3b, *P* = .014; N3a/ N3b, *P* = .048) was the only predictors for DM (Table 6).

**Table 4:** Multivariate analysis of predictors for locoregional recurrence in AGC patients after total gastrectomy.

Factor	Odds ratio	95% CI of odds ratio	*P*-value
T status^∗^			.114
T4a/T4b	0.292	0.108–0.785	**.015**
Type of lymphadenectomy			**.034**
D1/D2, D3	2.625	1.074–6.414	**.034**

**Table 5:** Multivariate analysis of predictors for peritoneal recurrence in AGC patients after total gastrectomy.

Factor	Odds ratio	95% CI of odds ratio	*P*-value
T status^∗^			.060
T4a/T4b	0.265	0.102–0.690	**.007**
Histological type			**.041**
Differentiated/Undifferentiated	0.121	0.016–0.920	**.041**

**Table 6:** Multivariate analysis of predictors for distant metastasis in AGC patients after total gastrectomy.

Factor	Odds ratio	95% CI of odds ratio	*P*-value
pN status^∗^			**.032**
N1/ N3b	0.073	0.009–0.581	**.013**
N2/ N3b	0.275	0.098–0.771	**.014**
N3a/ N3b	0.384	0.148–0.993	**.048**

## 4 Discussion

In China, the majority of GC patients were already in the advanced stage of disease at the time of diagnosis for lack of an effective GC screening system, resulting in a high rate of recurrence and relatively poor prognosis compared with Japan and South Korea.^[14–16]^ Although a more radical standardized surgical approach to treatment involving systematic lymph node dissection is considered to have contributed to better survival outcomes, recurrence after surgery does still occur in a considerable proportion of cases.^[17]^ Compared with partial gastrectomy, total gastrectomy not only resects the whole stomach, but also dissects a wider range of lymph nodes according to the latest Japanese gastric cancer treatment guidelines.^[18]^ When patients with large tumor size, tumor located in the upper stomach, or a tumor invaded neighboring organs which can be performed combined organ ressection, in order to ensure the negative status of surgical margins, a total gastrectomy is a rational choice. Patients with all these tumors have generally poor prognosis due largely to their special anatomical location and poor biological behavior. We retrospectively analysis the time and patterns of recurrence for AGC patients after curative total gastrectomy and further reveal predictive factors associated with each pattern of recurrence.

The recurrence pattern varies considerably between institutions and countries. It tends to be locoregional in Western countries while peritoneal in East.^[19]^ This maybe due to different extent of lymphadenectomy, most Western surgeons perform D1 lymphadenectomy while D2 dissection is widely used as a standard procedure for the treatment of GC in Eastern countries. However, DM was also regarded as the most frequent pattern by several literatures. For instance, Rohatgi et al^[3]^ found that DM (65.6%) was the most frequent recurrence pattern. Another Japanese study also reported DM (54%) was the most common pattern in GC patients after curative dissection.^[20]^ It was reported^[9,13]^ that recurrence patterns also impacted by tumor staging and location. The most common recurrence pattern of early stage GC is DM, while PR is the most common pattern in AGC.^[4]^ Youn et al^[7]^ reported DM (55.7%) was the most common pattern, followed by LR (34%) and PR (10.3%) in early disease. In node-negative GC patients, they were LR (54.1%), DM (36.5%), and PR (32.9%).^[8]^ For proximal GC,^[9]^ the most frequent pattern after curative surgery was LR (37%), then DM (35.5%) and PR (23.7%). In this study, DM (42.6%) was the first pattern, followed by peritoneal (36.8%) and locoregional recurrence (33.8%). Performing total gastrectomy may avoid recurrence of gastric remnant, reducing the incidence of LR. Recurrence occurred in 10% to 90% of GC patients within 2 years after surgery.^[6]^ While, the study of the relationship between recurrence rate and time of gastric cancer after total gastrectomy is rarely reported in literatures. In our study, 68 GC patients had postoperative recurrence, among them, 53 (77.9%) had recurrences within the first 2 years, 66 (97.1%) patients within 5 years after surgery. DM was the most common recurrence pattern within 2 years after operation, and the incidence of LR, especially remnant gastric cancer, increased significantly after 5 years. Therefore, follow-up plan should be made according to the recurrence time and the key point should be focused on the first 2 years after surgery.

There are many clinical and pathological factors related to the recurrence of GC patients, such as tumor size, lesion location, depth of invasion, lymph node metastasis, Lauren classification, and so on. In our study, type of lymphadenectomy, pT stage, pN stage, and number of negative lymph nodes were risk factors for recurrence of AGC after curative total gastrectomy by univariate analyses. Among them, pT stage and pN stage have been confirmed to be associated with postoperative recurrence and poor prognosis.^[21,22]^ Yokoyama et al^[13]^ confirmed that undifferentiated tumor is the only risk factor for recurrence of Ib staging patients regardless of the initial tumor depth and lymph nodes metastasis. Above all, the effect of the count of negative node on postoperative recurrence was mainly due to the high rate of lymph node metastases.^[23]^ In negative lymph nodes identified by general pathological examination, about 10% to 20% had lymph node micrometastases.^[24,25]^ These patients with lymph node micrometastases often have a higher probability of recurrence.^[25]^ Therefore, intraoperative detection of sufficient number of lymph nodes will reduce the potentially risk of recurrence.

Based on our results, in AGC patients following curative total gastrectomy, the recurrent factors differed significantly between locoregional/peritoneal recurrence and DM. T4b status and extent of lymphadenectomy were 2 independent factors for LR. Whether the extended lymph node dissection contributes to lower LR rate continues to be debated between East and West. Macdonald et al^[26]^ reported that the LR rates of radical resection group and surgery plus postoperative chemotherapy group were 29% and 19%, respectively, which changed the standard of treatment of resectable gastric cancer in the USA. However, 54% patients underwent D0 dissection and 36% patients underwent D1 dissection in their study, therefore, their results could not explain whether D2 surgery decrease LR rate. Several East series have reported a LR rate ranging from 8% to 19% following at least D2 dissection.^[9,16,19,21]^ Another Italian study proved that D2 dissection could be completed safely and allowed low LR rate (17.1%). In current study, the LR rate was 6% in patients following extended lymphadenectomy versus 16% in patients after limited lymphadenectomy. PR usually occurred in AGC patients with poor tissue differentiation type, and was relevant to serosal invasion and lymph node metastasis.^[20]^ In these cases, tumor cells invade the gastric wall and infiltrate the serosa, enter the abdominal cavity, peristalsis of the gastrointestinal, and other activities lead to the widespread spread of tumor cells. These peritoneal free cancer cells cause peritoneal metastasis. Moreover, poorly differentiated tumor cells are more likely to penetrate tissue. Similar to their findings, our results identified T4b status and undifferentiated tumor as 2 independent predictors for PR. DM was the domain recurrence pattern in this study with the pN staging as the only recurrent risk factor. That maybe due to the probability of microvascular invasion increase with advancing pN staging, these tumor cells into blood system may cause DM. Moreover, it was reported that patients with DM fared worse than patients with locoregional or peritoneal recurrence in terms of survival. Therefore, early detection and timely treatment are essential for patients with DM. According to what we already know, this study firstly reported the recurrence pattern of AGC following curative total gastrectomy. The flaw in our study is that the number of patients enrolled is too small, further researches with large samples are necessary to clarify predictors for each pattern of recurrence.

In conclusion, the present study reveals that clinicopathological factors of AGC determine the type of recurrence. T4b status was an independent risk factor for locoregional and peritoneal recurrence, undifferentiated tumors were more likely to recur in peritoneum, pN status is the only independent risk factor for DM, and extended lymphadenectomy was considered to decrease LR. Predictors for each type of recurrence could be used to guide postoperative follow-up and adjuvant treatment.

## Author contributions

**Conceptualization:** Xuguang Jiao.

**Data curation:** Yu Wang.

**Formal analysis:** Yu Wang.

**Investigation:** Xuguang Jiao, Xinbo Wang.

**Methodology:** Xuguang Jiao, Feng Wang.

**Resources:** Feng Wang.

**Software:** Yu Wang.

**Writing – original draft:** Xuguang Jiao.

**Writing – review & editing:** Feng Wang, Xinbo Wang.
